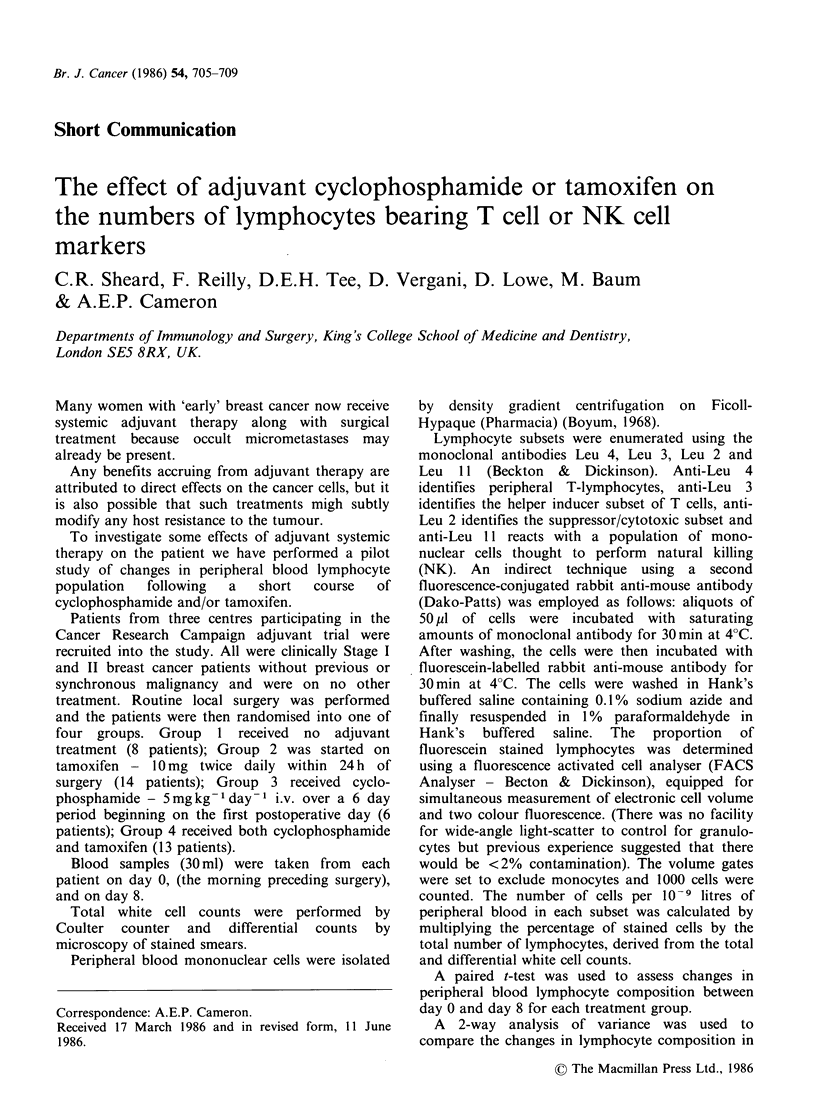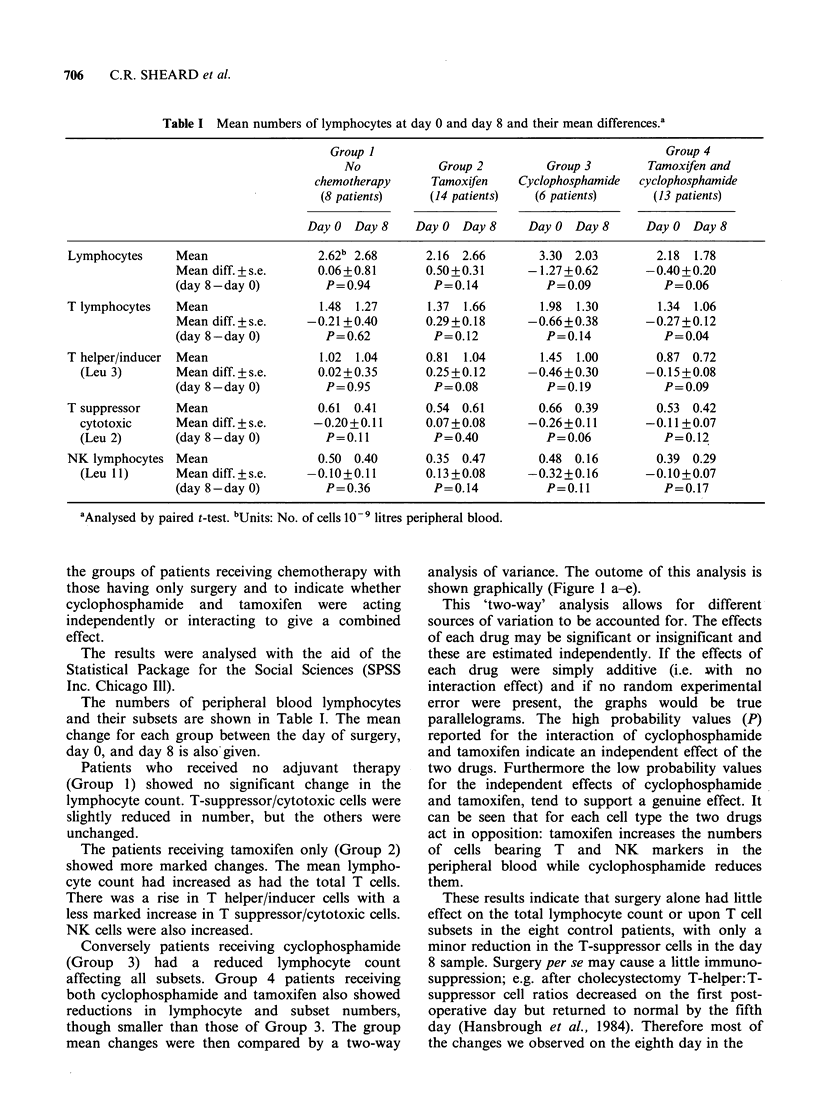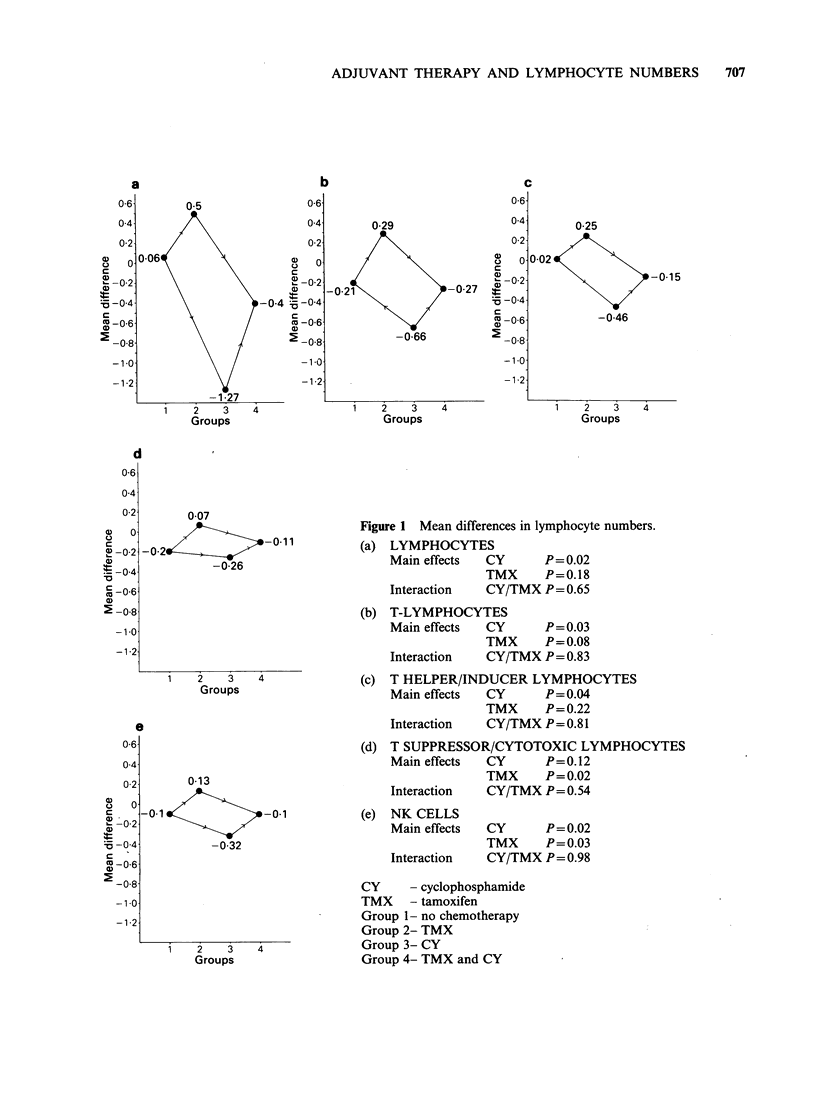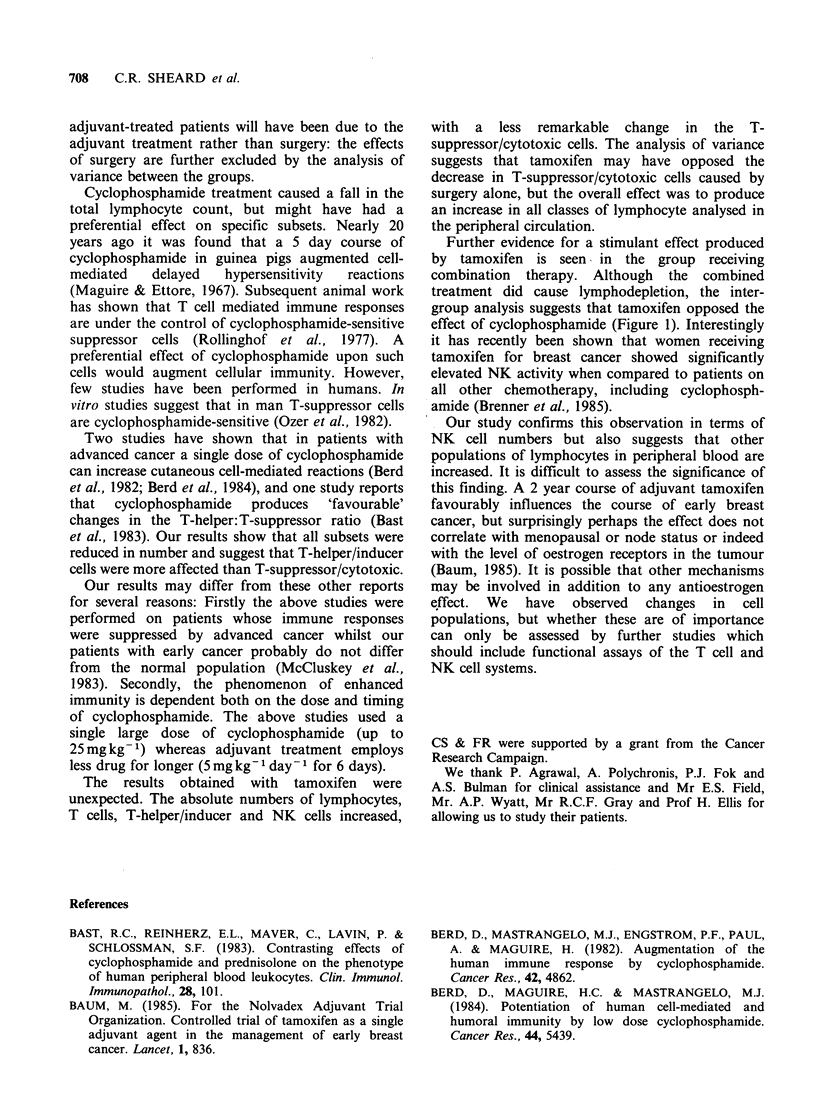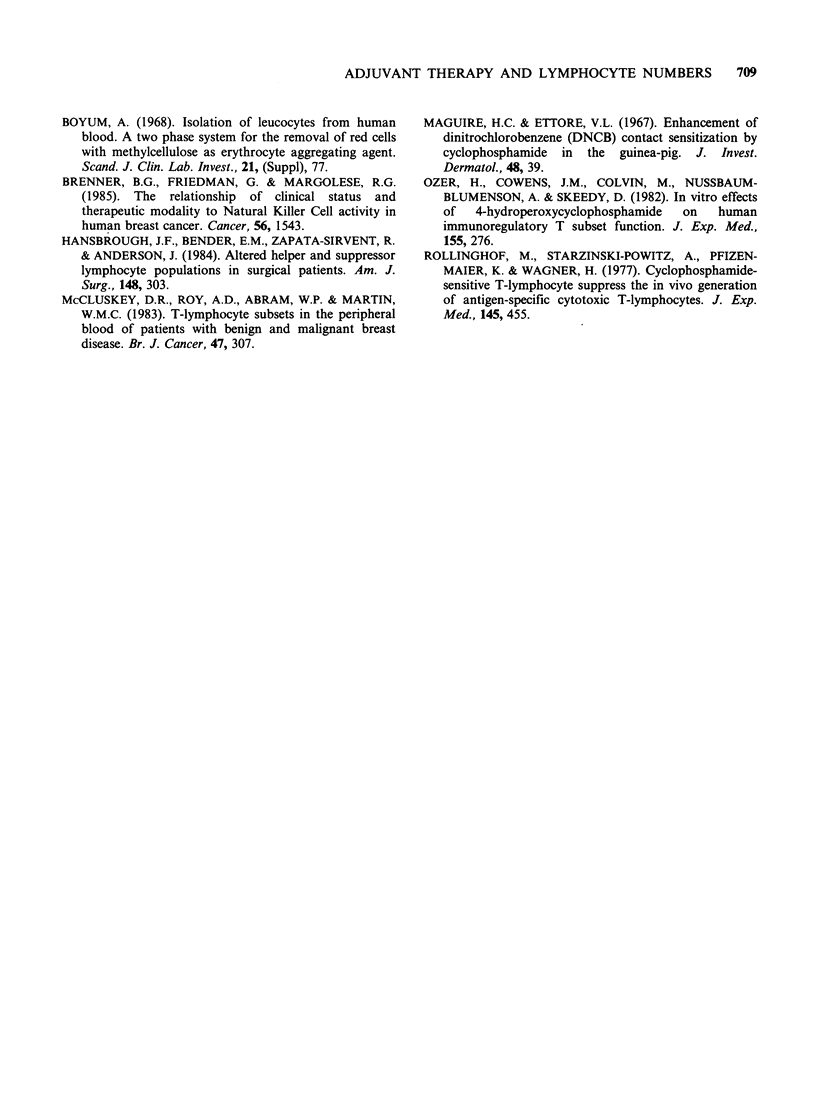# The effect of adjuvant cyclophosphamide or tamoxifen on the numbers of lymphocytes bearing T cell or NK cell markers.

**DOI:** 10.1038/bjc.1986.230

**Published:** 1986-10

**Authors:** C. R. Sheard, F. Reilly, D. E. Tee, D. Vergani, D. Lowe, M. Baum, A. E. Cameron


*Br. J. Cancer* (1986) **54**, 705–709

### Short Communication

### The effect of adjuvant cyclophosphamide or tamoxifen on the numbers of lymphocytes bearing T cell or NK cell markers

C.R. Sheard, F. Reilly, D.E.H. Tee, D. Vergani, D. Lowe, M. Baum & A.E.P. Cameron

*Departments of Immunology and Surgery, King's College School of Medicine and Dentistry, London SE5 8RX, UK.*

Many women with 'early' breast cancer now receive systemic adjuvant therapy along with surgical treatment because occult micrometastases may already be present.

Any benefits accruing from adjuvant therapy are attributed to direct effects on the cancer cells, but it is also possible that such treatments migh subtly modify any host resistance to the tumour.

To investigate some effects of adjuvant systemic therapy on the patient we have performed a pilot study of changes in peripheral blood lymphocyte population following a short course of cyclophosphamide and/or tamoxifen.

Patients from three centres participating in the Cancer Research Campaign adjuvant trial were recruited into the study. All were clinically Stage I and II breast cancer patients without previous or synchronous malignancy and were on no other treatment. Routine local surgery was performed and the patients were then randomised into one of four groups. Group 1 received no adjuvant treatment (8 patients); Group 2 was started on tamoxifen – 10 mg twice daily within 24 h of surgery (14 patients); Group 3 received cyclophosphamide – 5 mg kg$^{-1}$ day$^{-1}$ i.v. over a 6 day period beginning on the first postoperative day (6 patients); Group 4 received both cyclophosphamide and tamoxifen (13 patients).

Blood samples (30 ml) were taken from each patient on day 0, (the morning preceding surgery), and on day 8.

Total white cell counts were performed by Coulter counter and differential counts by microscopy of stained smears.

Peripheral blood mononuclear cells were isolated by density gradient centrifugation on Ficoll-Hypaque (Pharmacia) (Boyum, 1968).

Lymphocyte subsets were enumerated using the monoclonal antibodies Leu 4, Leu 3, Leu 2 and Leu 11 (Beckton & Dickinson). Anti-Leu 4 identifies peripheral T-lymphocytes, anti-Leu 3 identifies the helper inducer subset of T cells, anti-Leu 2 identifies the suppressor/cytotoxic subset and anti-Leu 11 reacts with a population of mononuclear cells thought to perform natural killing (NK). An indirect technique using a second fluorescence-conjugated rabbit anti-mouse antibody (Dako-Patts) was employed as follows: aliquots of 50 $\mu$l of cells were incubated with saturating amounts of monoclonal antibody for 30 min at 4°C. After washing, the cells were then incubated with fluorescein-labelled rabbit anti-mouse antibody for 30 min at 4°C. The cells were washed in Hank's buffered saline containing 0.1% sodium azide and finally resuspended in 1% paraformaldehyde in Hank's buffered saline. The proportion of fluorescein stained lymphocytes was determined using a fluorescence activated cell analyser (FACS Analyser – Becton & Dickinson), equipped for simultaneous measurement of electronic cell volume and two colour fluorescence. (There was no facility for wide-angle light-scatter to control for granulocytes but previous experience suggested that there would be $<2\%$ contamination). The volume gates were set to exclude monocytes and 1000 cells were counted. The number of cells per $10^{-9}$ litres of peripheral blood in each subset was calculated by multiplying the percentage of stained cells by the total number of lymphocytes, derived from the total and differential white cell counts.

A paired *t*-test was used to assess changes in peripheral blood lymphocyte composition between day 0 and day 8 for each treatment group.

A 2-way analysis of variance was used to compare the changes in lymphocyte composition in

Correspondence: A.E.P. Cameron.
Received 17 March 1986 and in revised form, 11 June 1986.

© The Macmillan Press Ltd., 1986

**Table I** Mean numbers of lymphocytes at day 0 and day 8 and their mean differences.[a]

| | | *Group 1 No chemotherapy (8 patients)* | | *Group 2 Tamoxifen (14 patients)* | | *Group 3 Cyclophosphamide (6 patients)* | | *Group 4 Tamoxifen and cyclophosphamide (13 patients)* | |
|---|---|---|---|---|---|---|---|---|---|
| | | *Day 0* | *Day 8* | *Day 0* | *Day 8* | *Day 0* | *Day 8* | *Day 0* | *Day 8* |
| Lymphocytes | Mean | 2.62[b] | 2.68 | 2.16 | 2.66 | 3.30 | 2.03 | 2.18 | 1.78 |
| | Mean diff. ± s.e. | 0.06 ± 0.81 | | 0.50 ± 0.31 | | −1.27 ± 0.62 | | −0.40 ± 0.20 | |
| | (day 8 − day 0) | $P = 0.94$ | | $P = 0.14$ | | $P = 0.09$ | | $P = 0.06$ | |
| T lymphocytes | Mean | 1.48 | 1.27 | 1.37 | 1.66 | 1.98 | 1.30 | 1.34 | 1.06 |
| | Mean diff. ± s.e. | −0.21 ± 0.40 | | 0.29 ± 0.18 | | −0.66 ± 0.38 | | −0.27 ± 0.12 | |
| | (day 8 − day 0) | $P = 0.62$ | | $P = 0.12$ | | $P = 0.14$ | | $P = 0.04$ | |
| T helper/inducer (Leu 3) | Mean | 1.02 | 1.04 | 0.81 | 1.04 | 1.45 | 1.00 | 0.87 | 0.72 |
| | Mean diff. ± s.e. | 0.02 ± 0.35 | | 0.25 ± 0.12 | | −0.46 ± 0.30 | | −0.15 ± 0.08 | |
| | (day 8 − day 0) | $P = 0.95$ | | $P = 0.08$ | | $P = 0.19$ | | $P = 0.09$ | |
| T suppressor cytotoxic (Leu 2) | Mean | 0.61 | 0.41 | 0.54 | 0.61 | 0.66 | 0.39 | 0.53 | 0.42 |
| | Mean diff. ± s.e. | −0.20 ± 0.11 | | 0.07 ± 0.08 | | −0.26 ± 0.11 | | −0.11 ± 0.07 | |
| | (day 8 − day 0) | $P = 0.11$ | | $P = 0.40$ | | $P = 0.06$ | | $P = 0.12$ | |
| NK lymphocytes (Leu 11) | Mean | 0.50 | 0.40 | 0.35 | 0.47 | 0.48 | 0.16 | 0.39 | 0.29 |
| | Mean diff. ± s.e. | −0.10 ± 0.11 | | 0.13 ± 0.08 | | −0.32 ± 0.16 | | −0.10 ± 0.07 | |
| | (day 8 − day 0) | $P = 0.36$ | | $P = 0.14$ | | $P = 0.11$ | | $P = 0.17$ | |

[a]Analysed by paired *t*-test. [b]Units: No. of cells $10^{-9}$ litres peripheral blood.

the groups of patients receiving chemotherapy with those having only surgery and to indicate whether cyclophosphamide and tamoxifen were acting independently or interacting to give a combined effect.

The results were analysed with the aid of the Statistical Package for the Social Sciences (SPSS Inc. Chicago Ill).

The numbers of peripheral blood lymphocytes and their subsets are shown in Table I. The mean change for each group between the day of surgery, day 0, and day 8 is also given.

Patients who received no adjuvant therapy (Group 1) showed no significant change in the lymphocyte count. T-suppressor/cytotoxic cells were slightly reduced in number, but the others were unchanged.

The patients receiving tamoxifen only (Group 2) showed more marked changes. The mean lymphocyte count had increased as had the total T cells. There was a rise in T helper/inducer cells with a less marked increase in T suppressor/cytotoxic cells. NK cells were also increased.

Conversely patients receiving cyclophosphamide (Group 3) had a reduced lymphocyte count affecting all subsets. Group 4 patients receiving both cyclophosphamide and tamoxifen also showed reductions in lymphocyte and subset numbers, though smaller than those of Group 3. The group mean changes were then compared by a two-way analysis of variance. The outome of this analysis is shown graphically (Figure 1 a–e).

This 'two-way' analysis allows for different sources of variation to be accounted for. The effects of each drug may be significant or insignificant and these are estimated independently. If the effects of each drug were simply additive (i.e. with no interaction effect) and if no random experimental error were present, the graphs would be true parallelograms. The high probability values (*P*) reported for the interaction of cyclophosphamide and tamoxifen indicate an independent effect of the two drugs. Furthermore the low probability values for the independent effects of cyclophosphamide and tamoxifen, tend to support a genuine effect. It can be seen that for each cell type the two drugs act in opposition: tamoxifen increases the numbers of cells bearing T and NK markers in the peripheral blood while cyclophosphamide reduces them.

These results indicate that surgery alone had little effect on the total lymphocyte count or upon T cell subsets in the eight control patients, with only a minor reduction in the T-suppressor cells in the day 8 sample. Surgery *per se* may cause a little immunosuppression; e.g. after cholecystectomy T-helper:T-suppressor cell ratios decreased on the first post-operative day but returned to normal by the fifth day (Hansbrough *et al.*, 1984). Therefore most of the changes we observed on the eighth day in the

**Figure 1** Mean differences in lymphocyte numbers.

(a) LYMPHOCYTES

| | | |
|---|---|---|
| Main effects | CY | $P=0.02$ |
| | TMX | $P=0.18$ |
| Interaction | CY/TMX | $P=0.65$ |

(b) T-LYMPHOCYTES

| | | |
|---|---|---|
| Main effects | CY | $P=0.03$ |
| | TMX | $P=0.08$ |
| Interaction | CY/TMX | $P=0.83$ |

(c) T HELPER/INDUCER LYMPHOCYTES

| | | |
|---|---|---|
| Main effects | CY | $P=0.04$ |
| | TMX | $P=0.22$ |
| Interaction | CY/TMX | $P=0.81$ |

(d) T SUPPRESSOR/CYTOTOXIC LYMPHOCYTES

| | | |
|---|---|---|
| Main effects | CY | $P=0.12$ |
| | TMX | $P=0.02$ |
| Interaction | CY/TMX | $P=0.54$ |

(e) NK CELLS

| | | |
|---|---|---|
| Main effects | CY | $P=0.02$ |
| | TMX | $P=0.03$ |
| Interaction | CY/TMX | $P=0.98$ |

CY – cyclophosphamide
TMX – tamoxifen
Group 1 – no chemotherapy
Group 2 – TMX
Group 3 – CY
Group 4 – TMX and CY

adjuvant-treated patients will have been due to the adjuvant treatment rather than surgery: the effects of surgery are further excluded by the analysis of variance between the groups.

Cyclophosphamide treatment caused a fall in the total lymphocyte count, but might have had a preferential effect on specific subsets. Nearly 20 years ago it was found that a 5 day course of cyclophosphamide in guinea pigs augmented cell-mediated delayed hypersensitivity reactions (Maguire & Ettore, 1967). Subsequent animal work has shown that T cell mediated immune responses are under the control of cyclophosphamide-sensitive suppressor cells (Rollinghof *et al.*, 1977). A preferential effect of cyclophosphamide upon such cells would augment cellular immunity. However, few studies have been performed in humans. *In vitro* studies suggest that in man T-suppressor cells are cyclophosphamide-sensitive (Ozer *et al.*, 1982).

Two studies have shown that in patients with advanced cancer a single dose of cyclophosphamide can increase cutaneous cell-mediated reactions (Berd *et al.*, 1982; Berd *et al.*, 1984), and one study reports that cyclophosphamide produces 'favourable' changes in the T-helper:T-suppressor ratio (Bast *et al.*, 1983). Our results show that all subsets were reduced in number and suggest that T-helper/inducer cells were more affected than T-suppressor/cytotoxic.

Our results may differ from these other reports for several reasons: Firstly the above studies were performed on patients whose immune responses were suppressed by advanced cancer whilst our patients with early cancer probably do not differ from the normal population (McCluskey *et al.*, 1983). Secondly, the phenomenon of enhanced immunity is dependent both on the dose and timing of cyclophosphamide. The above studies used a single large dose of cyclophosphamide (up to $25\,mg\,kg^{-1}$) whereas adjuvant treatment employs less drug for longer ($5\,mg\,kg^{-1}\,day^{-1}$ for 6 days).

The results obtained with tamoxifen were unexpected. The absolute numbers of lymphocytes, T cells, T-helper/inducer and NK cells increased, with a less remarkable change in the T-suppressor/cytotoxic cells. The analysis of variance suggests that tamoxifen may have opposed the decrease in T-suppressor/cytotoxic cells caused by surgery alone, but the overall effect was to produce an increase in all classes of lymphocyte analysed in the peripheral circulation.

Further evidence for a stimulant effect produced by tamoxifen is seen in the group receiving combination therapy. Although the combined treatment did cause lymphodepletion, the inter-group analysis suggests that tamoxifen opposed the effect of cyclophosphamide (Figure 1). Interestingly it has recently been shown that women receiving tamoxifen for breast cancer showed significantly elevated NK activity when compared to patients on all other chemotherapy, including cyclophosphamide (Brenner *et al.*, 1985).

Our study confirms this observation in terms of NK cell numbers but also suggests that other populations of lymphocytes in peripheral blood are increased. It is difficult to assess the significance of this finding. A 2 year course of adjuvant tamoxifen favourably influences the course of early breast cancer, but surprisingly perhaps the effect does not correlate with menopausal or node status or indeed with the level of oestrogen receptors in the tumour (Baum, 1985). It is possible that other mechanisms may be involved in addition to any antioestrogen effect. We have observed changes in cell populations, but whether these are of importance can only be assessed by further studies which should include functional assays of the T cell and NK cell systems.

CS & FR were supported by a grant from the Cancer Research Campaign.

We thank P. Agrawal, A. Polychronis, P.J. Fok and A.S. Bulman for clinical assistance and Mr E.S. Field, Mr. A.P. Wyatt, Mr R.C.F. Gray and Prof H. Ellis for allowing us to study their patients.

### References

BAST, R.C., REINHERZ, E.L., MAVER, C., LAVIN, P. & SCHLOSSMAN, S.F. (1983). Contrasting effects of cyclophosphamide and prednisolone on the phenotype of human peripheral blood leukocytes. *Clin. Immunol. Immunopathol.*, **28**, 101.

BAUM, M. (1985). For the Nolvadex Adjuvant Trial Organization. Controlled trial of tamoxifen as a single adjuvant agent in the management of early breast cancer. *Lancet*, **1**, 836.

BERD, D., MASTRANGELO, M.J., ENGSTROM, P.F., PAUL, A. & MAGUIRE, H. (1982). Augmentation of the human immune response by cyclophosphamide. *Cancer Res.*, **42**, 4862.

BERD, D., MAGUIRE, H.C. & MASTRANGELO, M.J. (1984). Potentiation of human cell-mediated and humoral immunity by low dose cyclophosphamide. *Cancer Res.*, **44**, 5439.

BOYUM, A. (1968). Isolation of leucocytes from human blood. A two phase system for the removal of red cells with methylcellulose as erythrocyte aggregating agent. *Scand. J. Clin. Lab. Invest.*, **21**, (Suppl), 77.

BRENNER, B.G., FRIEDMAN, G. & MARGOLESE, R.G. (1985). The relationship of clinical status and therapeutic modality to Natural Killer Cell activity in human breast cancer. *Cancer*, **56**, 1543.

HANSBROUGH, J.F., BENDER, E.M., ZAPATA-SIRVENT, R. & ANDERSON, J. (1984). Altered helper and suppressor lymphocyte populations in surgical patients. *Am. J. Surg.*, **148**, 303.

McCLUSKEY, D.R., ROY, A.D., ABRAM, W.P. & MARTIN, W.M.C. (1983). T-lymphocyte subsets in the peripheral blood of patients with benign and malignant breast disease. *Br. J. Cancer*, **47**, 307.

MAGUIRE, H.C. & ETTORE, V.L. (1967). Enhancement of dinitrochlorobenzene (DNCB) contact sensitization by cyclophosphamide in the guinea-pig. *J. Invest. Dermatol.*, **48**, 39.

OZER, H., COWENS, J.M., COLVIN, M., NUSSBAUM-BLUMENSON, A. & SKEEDY, D. (1982). In vitro effects of 4-hydroperoxycyclophosphamide on human immunoregulatory T subset function. *J. Exp. Med.*, **155**, 276.

ROLLINGHOF, M., STARZINSKI-POWITZ, A., PFIZEN-MAIER, K. & WAGNER, H. (1977). Cyclophosphamide-sensitive T-lymphocyte suppress the in vivo generation of antigen-specific cytotoxic T-lymphocytes. *J. Exp. Med.*, **145**, 455.